# Dissecting mechanisms of brain aging by studying the intrinsic excitability of neurons

**DOI:** 10.3389/fnagi.2014.00337

**Published:** 2015-01-06

**Authors:** Valerio Rizzo, Jeffrey Richman, Sathyanarayanan V. Puthanveettil

**Affiliations:** Department of Neuroscience, The Scripps Research InstituteJupiter, FL, USA

**Keywords:** aging, action potential, single neuron, synaptic transmission, electrophysiology

## Abstract

Several studies using vertebrate and invertebrate animal models have shown aging associated changes in brain function. Importantly, changes in soma size, loss or regression of dendrites and dendritic spines and alterations in the expression of neurotransmitter receptors in specific neurons were described. Despite this understanding, how aging impacts intrinsic properties of individual neurons or circuits that govern a defined behavior is yet to be determined. Here we discuss current understanding of specific electrophysiological changes in individual neurons and circuits during aging.

## Introduction

Brain aging entails several changes in physiology and a progressive decline in cognitive functions (Burke and Barnes, 2006). However, cellular alterations related to this decline are not completely understood. Harold Brody (1955) postulated a decline in neuron number in all cortical layers during aging. Later investigations supported his work, showing a decline in cortical and subcortical neuron density (Ball, 1977; Coleman and Flood, 1987) of aging humans (Ball, 1977) and non-human primates (Brizzee and Knox, 1980). Nonetheless, in the 1980s, it was discovered that normal aging was not actually marked by neuronal death (West, 1993). Many studies showed that significant neuronal loss in the hippocampus (HP) and neocortex are not characteristics of normal aging in humans (West et al., 1994; Pakkenberg and Gundersen, 1997), non-human primates (Peters et al., 1994; Gazzaley et al., 1997; Merrill et al., 2000; Keuker et al., 2003) and rodents (Rapp and Gallagher, 1996; Rasmussen et al., 1996; Merrill et al., 2001).

**Figure 1:**
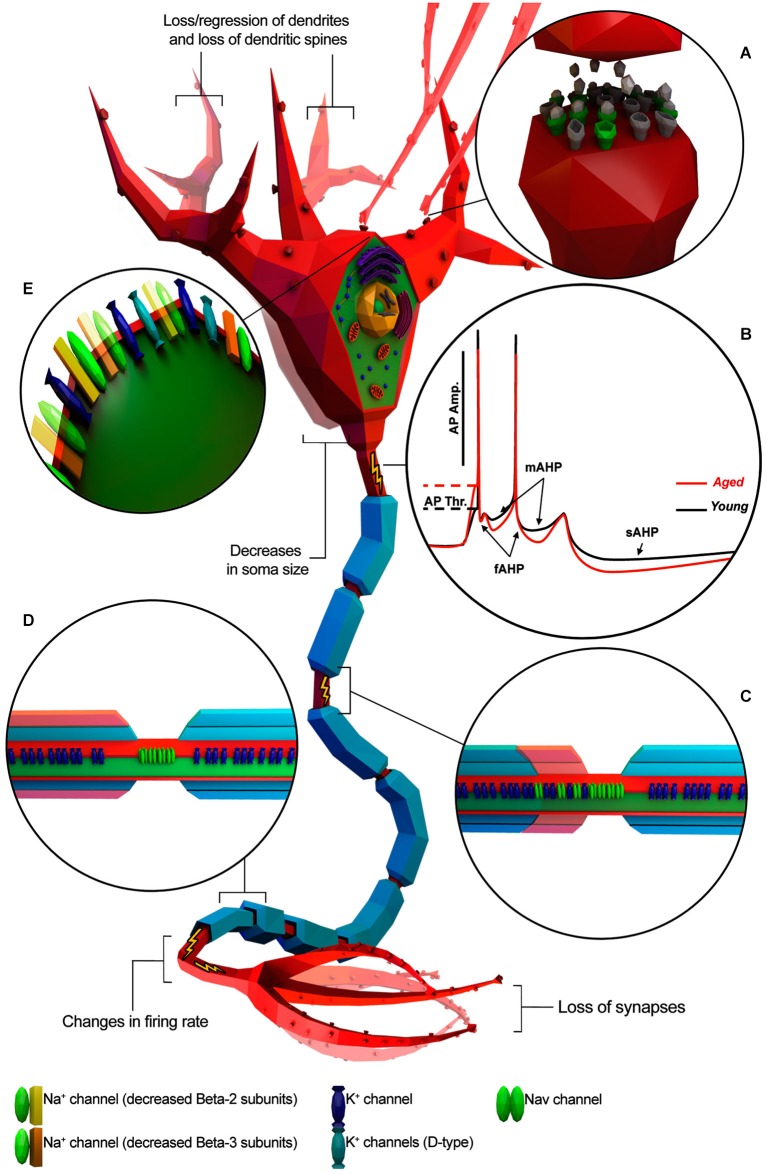
**Known age-related electrophysiological and morphological changes in neurons**. Neuron is depicted as a generic myelinated neuron for simplicity, in order to summarize information from different animal models and neuronal cell type. **(A)** Age-related increase of altered neurotransmitter receptors (gray) and decreased expression of normal ones (green). **(B)** Representative AP traces of young and aged neuron. **(C)** Absence/disruption of paranodal ultrastructure (in red) leading K^+^ channels to be relocated adjacent to “Na_v_” clusters. **(D)** Depletion of myelin layers (in red) leading to an increase of the occurrence of redundant myelin sheaths exposing the enclosed axon. **(E)** Alterations in Na^+^ and K^+^ channel properties or subtype expression patterns. (Illustration was created using Autodesk 3Ds Max and Adobe Photoshop).

Several studies support the hypothesis that cognitive deficits occurring in normal aging could be due to alterations in the intrinsic cellular properties of neurons (Driver et al., 2004; Chang et al., 2005; Wilson et al., 2005; Disterhoft and Oh, 2007; Luebke and Chang, 2007; Matthews et al., 2009). Investigations on the central nervous system (CNS) structures with crucial roles in cognitive processing have shown age-related alteration of intrinsic neuronal excitability (Landfield and Pitler, 1984; Disterhoft and Oh, 2007; Matthews et al., 2009; Oh et al., 2010; Wang et al., 2011). Consistent with this idea, aging neurons have been observed undergoing structural changes such as decreases in soma size (de Brabander et al., 1998; Wong et al., 2000; Figure 1), loss/regression of dendrites and loss of dendritic spines (Jacobs et al., 1997; Peters et al., 1998; Page et al., 2002; Duan et al., 2003; Figure 1), loss of synapses (Chen et al., 1995; Wong et al., 1998; Figure 1), alterations in neurotransmitter receptors (Post-Munson et al., 1994; Rosene and Nicholson, 1999; Figure 1) and/or decreased response to neurotransmitters (Fieber et al., 2010; Akhmedov et al., 2013; Kempsell and Fieber, 2014). Changes in neuronal physiology and structure lead to a less efficient transmission of information encoded in the form of action potentials (APs; Chang et al., 2005; Luebke and Chang, 2007) and impairment of the computational efficacy of the neuronal network (Randall et al., 2012). Taken together, these studies lead to three main questions: (1) Does normal aging impact electrophysiological properties of individual neurons? (2) Do morphological and molecular changes during aging correlate with electrophysiological changes? (3) Are these changes conserved across different species?

To address these questions, we should first understand whether and how aging affect the intrinsic electrophysiological properties of neurons and whether these changes affect neuronal communication and plasticity. We will first review our knowledge on changes in APs because APs play a central role in communication. APs are short-lasting events characterized by the rapid rise and fall of electrical membrane potential, which play a central role in encoding information in the form of specific frequency and pattern. Hence we will discuss changes in various parameters of APs during aging (Table 1; Figure 1).

**Table 1:** **Aging associated changes in action potentials (APs)**.

Species	AP Threshold	AP Amplitude	AHP	AP Conduction	AP Firing	References
**Monkey**	–No change in Layer 2/3 PFC-PC;	–Decrease in layer 2/3 PFC-PC;	–Increase in Layer 2/3 PFC-PC;	na	–Increase in Layer 2/3 PFC-PC;	Schmolesky et al. (2000), Leventhal et al. (2003), Luebke and Rosene (2003), Chang et al. (2005), Luebke and Chang (2007), Luebke et al. (2013)
	–No change in Layer 5 PFC-PC;	–Decrease in Layer 5 PFC-PC;	–Increase in Layer 5 PFC-PC;		–Increase in Layer 5 PFC-PC;		
	–No change in Layer 3 V1-PC;	–No change in Layer 3 V1-PC;	–No change in Layer 3 V1-PC;		–Increase in layer 3 V1-PC		
	–No change in DGCL	–No change in DGCL	–No change in DGCL				
**Rat**	–Increase in HP-CA1-PC;	–No change in HP-CA1-PC;	–Increase in HP-CA1-PC;	–Decrease in Nucleus Basal is cortical afferent cholinergic neurons;	–Increase in HP-CA3-PIC;	Rogers et al. (1981), Landfield and Pitler (1984), Aston-Jones et al. (1985), Kerr et al. (1989), Hsu et al. (2002), Kumar and Foster (2004), Wilson et al. (2005), Gant et al. (2006), Matthews et al. (2009), Hickmott and Dinse (2013)
	–Increase in Layer 3 S1-PC;	–No change in Layer 3 S1-PC	–No change in Layer 3 S1-PC;	–Decrease in Parallel fiber Purkinje cell circuitry	–No change in HP-CA1-PIC		
**Mouse**	–Increase in HP-CA1-PC;	–No change in HP-CA1-PC;	–Increase in HP-CA1-PC;	na	–Decrease in HP-CA1-PC	Lu et al. (2011), Randall et al. (2012)
	–No change on HP-CA3-IN;	–No change in HP-CA3-IN	–No change in HP-CA3-IN		–Increase in HP-CA1-PIC		
**Rabbit**	–Increase in HP-CA1-PC	na	–Increase in HP-CA1-PC;	na	–Decrease in HP-CA1-PC	Moyer et al. (1992), Disterhoft et al. (1996), Moyer et al. (2000), Power et al. (2002), Disterhoft and Oh (2006), Disterhoft and Oh (2007)
			–No change in HP-CA3-IN				
**Aplysia**	–Increase in PVC-SN;	–Increase in PVC-SN;	na	–Decrease in R2	–Decrease in PVC-SN	Harley (1975), Akhmedov et al. (2013), Kempsell and Fieber (2014)
	–Increase in BSC-SN;	–Increase in BSC-SN;			–Decrease in BSC-SN		
	–No change in TMN;	–No change in TMN;			–No change in TMN		
	–No data on R15;	–No change in R15			–No change in R15		
**Cat**	na	na	na	–Decrease in spinal cord motoneurons;	-Increase in cat V1-PC	Chase et al. (1985), Morales et al. (1987), Xi et al. (1999)
				–Decrease in Pyramidal tract neurons			

### AP threshold

AP threshold is the critical level to which the membrane potential must be depolarized in order to initiate an AP and hence it is often used as a measure for neuron excitability. Recent studies have reported an age-related increase in the AP threshold of the rat hippocampal CA1 pyramidal cell (HP-CA1-PC; Matthews et al., 2009) and primary somatosensory cortex layer 3 pyramidal cell (S1-PC) (Hickmott and Dinse, 2013), mice (Randall et al., 2012) and rabbit HP-CA1-PC (Power et al., 2002) and also in the ventral pleural ganglion sensory neuron (PVC-SN) and buccal ganglion sensory neuron (BSC-SN) of aging *Aplysia* (Kempsell and Fieber, 2014). While the basis of this change has not yet been clarified, age-related depolarization of the AP threshold could likely be ascribed to alterations of voltage-gated Na^+^ channel (Na_v_ channel) activation properties or channel subtype expression patterns (Randall et al., 2012).

This age-related change in AP threshold dampens excitability of neurons and may affect neuronal activity by reducing transmission properties of neurons and their computing capability. Such an impairment of neuronal function in a brain region could constitute a functional lesion that may form the basis for cognitive decline during aging. Importantly, a direct correlation between the AP threshold and age-related learning or cognitive deficits is yet to be described (Matthews et al., 2009).

It is also important to consider that the above mentioned observations might not be universally true. For example, studies on age-related cognitive deficits in the Rhesus Monkey reported no change in the AP threshold in both layer 2/3 and 5 pyramidal cell of prefrontal cortex (PFC-PC; Luebke et al., 2013), layer 3 pyramidal cell of visual cortex 1 (V1-PC), dentate granule cell layer (DGCL; Luebke and Rosene, 2003), rat HP CA3 interneuron (HP-CA3-IN; Lu et al., 2011) and tail motoneuron (TMN) of aging *Aplysia* (Kempsell and Fieber, 2014). Taken together, these studies suggest that age-related changes in AP threshold may differentially affect species, neuron types, neuronal layers or sub regions of brain. Because of the lack of sufficient experimental data on the comparative analyses of changes in AP threshold in neurons from the different brain regions and correlation with specific functions, it is difficult to draw exhaustive conclusions of significance of the AP threshold during aging.

### AP amplitude

The amplitude of the AP plays a crucial role in evoking Ca^2+^ currents (I_Ca_) and the amount of neurotransmitter released by the axon terminals. Investigations in primates reported an age-related decrease of AP amplitude of PFC-PC in layer 2/3 and 5 (Chang et al., 2005; Luebke and Chang, 2007; Figure 1B). Such alterations in amplitude could be explained by either a reduction of Na^+^ channels and/or an increase in K^+^ channels involved in a D-type current. Indeed, numerous lines of evidence have suggested that age-related changes in AP amplitude could result from altered expression of “Na_v_” channel subunits, reduced expression of Na^+^ channels or altered expression of K^+^ channels involved in the K^+^ currents *I*_K_, *I*_A_, and *I*_D_ (Foehring and Surmeier, 1993; Korngreen and Sakmann, 2000; Chang et al., 2005; Luebke and Chang, 2007). Altered expression of voltage-gated delayed rectifier alpha Kv9.1 subunits seem to be particularly involved in age-related AP amplitude changes (Erraji-Benchekroun et al., 2005; Luebke and Chang, 2007), but further voltage-clamp studies would be required to gain a deeper insight.

Interestingly, normal aging has no effect on the AP properties of the aging monkey DGCL and layer 3 V1-PC (Luebke and Rosene, 2003; Luebke et al., 2013) suggesting different effects of aging on different populations of neurons (Luebke et al., 2004). This difference in findings is interesting since it could suggest that with regard to specific electrophysiological changes, specific populations of neurons might be more vulnerable to the effects of aging.

Similarly, no age-related change has been reported in the AP amplitude of the rat HP-CA1-PC and S1-PC (Gant et al., 2006; Matthews et al., 2009; Hickmott and Dinse, 2013), mice HP-CA1-PC and HP-CA3-IN (Lu et al., 2011; Randall et al., 2012) and *Aplysia* R15 neuron, PVC-SN, BSC-SN and TMN (Akhmedov et al., 2013; Kempsell and Fieber, 2014) suggesting that in these particular animal models the aging process may not be affecting normal resetting of “Na_v_” channels and/or opening of voltage-gated K^+^ channels. Interestingly, age-related change in AP amplitude does not seem to contribute to age-related cognitive decline or behavioral impairment (Luebke and Chang, 2007).

### After hyperpolarization (AHP)

AHP is the hyperpolarizing phase of AP hindering the membrane potential from reaching the threshold for generating a new AP or continuing firing activity (Madison and Nicoll, 1984; Lancaster and Nicoll, 1987; Storm, 1990; Sah, 1996). AHP is responsible for the regulation of excitability and may be essential for normal integration of neurotransmission. An increase in AHP has been postulated to be a factor in age-related learning impairments (Disterhoft and Oh, 2006, 2007; Foster, 2007).

AHP has three components (Figure 1B): (1) fast AHP (fAHP), medium AHP (mAHP), and slow AHP (sAHP) and each component is individually related to different K^+^ currents (Storm, 1990; Maccaferri et al., 1993; Sah, 1996; Stocker et al., 1999; Kumar and Foster, 2007). Indeed, studies on Kvβ1.1 mutant mice have suggested that age-related AHP increase and behavioral impairment could be a direct consequence of altered expression of specific K^+^ -channel subunits (Giese et al., 1998; Need et al., 2003). fAHP occurs after single APs whereas mAHP and sAHP occur after a high frequency burst of APs as is typically observed in normal HP-CA1-PC during learning and memory.

fAHP is mediated by the *I*_C_ K^+^ current, which contributes to the repolarization of AP (Shao et al., 1999; Sesti et al., 2010) whereas mAHP is mediated by the *I_AHP_* K^+^ current (Storm, 1990; Maccaferri et al., 1993; Sah, 1996; Stocker et al., 1999) and sAHP is mediated by *I_AHP_* K^+^ current (Lancaster and Adams, 1986; Sah, 1996; Stocker et al., 1999; Sah and Faber, 2002; Bond et al., 2004).

In general, several pieces of evidence have reported an increase in AHP amplitude and duration in HP-CA1-PC of rat (Landfield and Pitler, 1984; Kerr et al., 1989; Hsu et al., 2002; Kumar and Foster, 2004; Gant et al., 2006) and rabbit (Moyer et al., 1992, 2000; Disterhoft et al., 1996; Power et al., 2002) as well as in monkey Layer 2/3 and 5 PFC-PC during aging (Chang et al., 2005; Luebke and Chang, 2007; Figure 1B). Conversely, no change has been observed in AHP properties of the aging monkey DGCL (Luebke and Rosene, 2003) and layer 3 V1-PC (Luebke et al., 2013), in mice HP-CA3-IN (Lu et al., 2011) and rat S1-PC (Hickmott and Dinse, 2013) suggesting that alteration of the K^+^ currents may occur during aging in specific populations of neurons. Unfortunately, data on non-vertebrate animal models is not yet available for comparisons.

Examinations of the fAHP in aged animals revealed that alteration of this component is not common (Kumar and Foster, 2007; Matthews et al., 2009; Oh et al., 2010) whereas alteration of either mAHP or sAHP or both appear to be quite frequent during normal aging (Disterhoft and Oh, 2006; Kumar and Foster, 2007; Oh et al., 2010). Interestingly, simultaneous age-related change of both mAHP and sAHP has been observed in the rat (Gant et al., 2006) and rabbit HP-CA1-PC (Power et al., 2002) while Layer 2/3 and 5 PFC-PC of aged monkey show changes only in sAHP (Chang et al., 2005; Luebke and Chang, 2007).

mAHPs result from the activation of several K^+^ channels such as M-channels, H-channels, and Small conductance Ca^2+^-activated K^+^ channels (SK channels) (Stocker et al., 1999; Bond et al., 2004; Villalobos et al., 2004; Gu et al., 2005). Intriguingly, age-related upregulation of SK-channels in HP-CA1-PC have been observed to cause memory loss in mice (Dodge and Cooley, 1973) suggesting a direct involvement of this specific ion channel in age-related memory impairment. It remains to be determined whether age-related memory deficits may directly stem from changes in mAHP caused by altered expression of specific SK channels. On the other hand, age-related increase in sAHPs seems to be mediated by voltage-gated L-type Ca^2+^ channel (LTTC) and increased LTTC activity could be directly related to cognitive impairment since L-type calcium channel blockers has been demonstrated to ameliorate the age-related learning deficits, along with a reduction of AHP amplitude in aging rabbits (Disterhoft and Oh, 2006, 2007). It is likely that the increase in LTCC function underlying sAHP increased amplitude may be relatively specific to HP-CA1-PC since increased LTTC currents have only been observed in aged rat HP-CA1-PC (Campbell et al., 1996; Thibault and Landfield, 1996; Thibault et al., 2001; Brewer et al., 2009) while no age-related change in LTTC expression or activity within the cortex has been observed (Tanaka and Ando, 2001; Iwamoto et al., 2004).

### AP axonal conduction velocity

The flow of information within the nervous system relies on the AP conduction rate, which is determined by the passive and active flow of current. Several studies describe age-related decreases in axon conduction velocity in rat Nucleus Basalis cortical afferent cholinergic neurons (Aston-Jones et al., 1985) and parallel fiber Purkinje cell circuitry (Rogers et al., 1981) as well as in cat spinal cord motoneurons (Chase et al., 1985; Morales et al., 1987) and pyramidal neurons (Xi et al., 1999). Such age-related change in CNS seems to occur only in myelinated neurons since similar phenomena has not been reported in unmyelinated neurons in vertebrate or invertebrate animal models. Indeed, changes in the myelin sheath organization occurring in normal aging have been suggested as major contributors to age-related decrease in AP conduction (Peters et al., 1996; Peters, 2002). Interestingly such changes has been postulated in a study on the *Aplysia* R2 motoneuron (Harley, 1975).

Several studies have suggested that changes in myelin structure may affect conduction velocity by altering the location of specific ion channels on the axon (Figures 1C,D). For example, Hinman et al. (2006) have found that aging is associated with increased paranode disorganization, abnormalities in paranodal-juxtaparanodal junctions maintenance, and variation in paranodal ultrastructure (Hinman et al., 2006) resulting in an increased expression of K_v_1.2 channels in paranodal regions of the axon of both the monkey and rat optic nerve. These mislocalized K_v_ channels may compromise axonal function (Hinman et al., 2006) by stabilizing the membrane voltage (Rasband, 2004), thus impeding AP axonal conduction (Chiu, 1991). Also, changes in myelin ultrastructure increase the occurrence of redundant myelin sheaths exposing the enclosed axon (Peters et al., 2001; Peters, 2002) in the end reducing or interrupting saltatory AP axonal conduction (Hinman et al., 2006).

Alternatively, changes in myelin sheath organization seen in aging may also affect the expression of Na_v_ channels. Indeed, it has been demonstrated that lack of compact myelin structures in Shiverer mouse optic nerve leads to a diffuse expression of Na_v_1.2 channels and lower expression of Na_v_1.5 channels (Boiko et al., 2001). However, age-related alteration of axonal Na^+^_v_ channel localization in nodal structure and the putative consequences on AP axonal conduction velocity are not known.

Age-related changes in myelin structure and composition have been found in the rat corpus callosum (Sugiyama et al., 2002), in mice spiral ganglion neurons (SGNs; Xing et al., 2012), human (Albert, 1993), and non-human primate (Peters, 2002; Sloane et al., 2003). Even though age-related cognitive decline has been suggested to be a consequence of an alteration of the integrity of myelinated axons (Peters et al., 1996; Peters, 2002), whether they also correlate with a reduction in axonal conduction velocity is still unknown.

### Firing rate

Information in the nervous system is encoded and transmitted in the form of a specific pattern of APs. Neurons build these patterns by changing the firing rate of their APs. Several lines of evidence have reported age-related alteration of the AP firing rate in different animal models and a direct correlation between age-related alterations of AP firing rate and decline of cognitive functions or behavioral impairment (Chang et al., 2005; Wilson et al., 2005; Burke and Barnes, 2006; Caetano et al., 2012; Branch et al., 2014). Intriguingly, in monkeys differential aging process have been observed in layers 2/3 of PFC-PC that undergoes an increase in the AP firing rate (Chang et al., 2005) while layer 5 PFC-PC does not show any age-related changes in firing rate (Luebke and Chang, 2007).

Similarly, aging associated alteration of the HP AP firing rate has been observed to be sub region specific given that rat HP-CA3 place cell (HP-CA3-PlC) undergoes an increase of the AP firing rate during aging whereas HP-CA1-PlC does not (Wilson et al., 2005). Interestingly, age-related decrease in the AP firing rate has been reported in mouse HP-CA1-PC (Randall et al., 2012) whereas in mouse HP-CA1-PlC increase its AP firing rate during aging (Yan et al., 2003). Intriguingly, the *Aplysia* R15 neuron do not change their firing properties during aging (Akhmedov et al., 2013) while PVC-SN and BSC-SN decrease their AP firing rate (Kempsell and Fieber, 2014). These findings suggest that the aging process could differentially affect AP firing properties at the single neuron level.

Interestingly, other studies suggest conservation of mechanisms of aging in analogous brain regions of different animal models. For example, V1-PC show increased firing rate in both aged monkeys (Schmolesky et al., 2000; Leventhal et al., 2003; Chang et al., 2005) and cat (Hua et al., 2006) suggesting that some mechanisms could be shared by different species. Several of the age-related factors discussed in this review could have a direct impact on a neuron’s firing rate. For example, the change in neuron’s AP axonal conduction observed in aged monkeys and rat optic nerve (Hinman et al., 2006) could likely affect a neuron’s AP firing rate as well. Firing rate could also be altered by AHP whose age-related changes prolong the repolarization phase of APs and lengthen the period of time that neurons cannot fire new APs (Kumar and Foster, 2007; Sesti et al., 2010). For example, as discussed above increased Ca^2+^ influx via LTTC may cause an increase in the amplitude of sAHPs in rat HP-CA1-PC neurons (Kumar and Foster, 2007; Kumar et al., 2009) leading to a prolonged period of quiescence between AP bursts (Sesti et al., 2010). Importantly, a similar age-related increased Ca^2+^ influx could have the opposite effect on the AP firing rate in different brain regions. In fact, a recent study by Hickmott and Dinse (2013) have found that age-related increase in T-type Ca^2+^ currents in rat S1-PC is able to switch firing patterns to burst firing.

## Conclusion

We have discussed our current understanding of electrophysiological changes in individual neurons associated with aging (Table 1; Figure 1). We have also compared evidence from studies using different animal models wherever possible. It is important to note that aging affects fundamental properties of neurons, such as phases of APs, AP firing pattern and AP conduction velocity, leading to specific changes in neuronal communication and plasticity. However, age-related alterations of the intrinsic excitability of neurons might reflect secondary consequences of aging while others might be compensatory mechanisms to such changes. For example, the decrease in excitatory synaptic transmission and increase in inhibitory synaptic transmission in layer 2/3 PFC-PC (Luebke et al., 2004) seen in aged monkeys could, hypothetically, represent a compensatory response to significantly increased AP firing rates observed in layer 2/3 PFC-PC of the aged monkey (Chang et al., 2005). Consistent with the gene expression data on single neurons (Moroz and Kohn, 2010; Kadakkuzha et al., 2013) aging associated changes in electrophysiological properties are mostly specific to species, brain region and neuronal type. It remains to be shown whether specific electrophysiological changes in neurons directly lead to a behavioral deficit during aging. Further integrated approaches that combine behavioral, electrophysiological and genomic analysis of individual neurons and circuits might help solve this challenging problem. Lower organisms such as the snail *Aplysia* where fewer neurons regulate specific behavior such as gill withdrawal reflex is ideally suited for the gain of function experiments to address this challenge.

## Conflict of interest statement

The authors declare that the research was conducted in the absence of any commercial or financial relationships that could be construed as a potential conflict of interest.

## Abbreviations

HP: Hippocampus
CNS: Central Nervous System
APs: Action Potentials
S1-PC: Primary somatosensory cortex pyramidal cell
CA1: Cornus Ammonis 1
CA3: Cornus Ammonis 3
HP-CA1-PC: Hippocampal CA1 pyramidal cell
HP-CA3-PC: Hippocampal CA3 pyramidal cell
HP-CA3-IN: Hippocampal CA3 interneuron
HP-CA3-PlC: Hippocampal CA3 place cell
HP-CA1-PlC: Hippocampal CA1 place cell
PVC-SN: Ventral pleural ganglion sensory neuron
BSC-SN: Buccal ganglion sensory neuron
PFC-PC: Prefrontal cortex pyramidal cell
V1-PC: Visual cortex pyramidal cell
DGCL: Dentate granule cell layer
AHP: Afterhyperpolarization
fAHP: Fast Afterhyperpolarization
mAHP: Medium Afterhyperpolarization
sAHP: Slow Afterhyperpolarization
LTTC: L-type Ca^2+^ channel
Na_v_ channel: Voltage-gated Na^+^ channel
SGNs: Spiral ganglion neurons.

## References

[B1] Akhmedov K. Rizzo V. Kadakkuzha B. M. Carter C. J. Magoski N. S. Capo T. R.. (2013). Decreased response to acetylcholine during aging of aplysia neuron R15. PLoS One 8:e84793. 10.1371/journal.pone.0084793 24386417 PMC3874043

[B2] Albert M. (1993). Neuropsychological and neurophysiological changes in healthy adult humans across the age range. Neurobiol. Aging 14, 623–625. 10.1016/0197-4580(93)90049-h 8295666

[B3] Aston-Jones G. Rogers J. Shaver R. D. Dinan T. G. Moss D. E. (1985). Age-impaired impulse flow from nucleus basalis to cortex. Nature 318, 462–464. 10.1038/318462a0 4069217

[B4] Ball M. J. (1977). Neuronal loss, neurofibrillary tangles and granulovacuolar degeneration in the hippocampus with ageing and dementia. A quantitative study. Acta Neuropathol. 37, 111–118. 10.1007/bf00692056 848276

[B5] Boiko T. Rasband M. N. Levinson S. R. Caldwell J. H. Mandel G. Trimmer J. S.. (2001). Compact myelin dictates the differential targeting of two sodium channel isoforms in the same axon. Neuron 30, 91–104. 10.1016/s0896-6273(01)00265-3 11343647

[B6] Bond C. T. Herson P. S. Strassmaier T. Hammond R. Stackman R. Maylie J.. (2004). Small conductance Ca2+-activated K+ channel knock-out mice reveal the identity of calcium-dependent afterhyperpolarization currents. J. Neurosci. 24, 5301–5306. 10.1523/jneurosci.0182-04.2004 15190101 PMC2831645

[B7] Branch S. Y. Sharma R. Beckstead M. J. (2014). Aging decreases L-type calcium channel currents and pacemaker firing fidelity in substantia nigra dopamine neurons. J. Neurosci. 34, 9310–9318. 10.1523/JNEUROSCI.4228-13.2014 25009264 PMC4087208

[B8] Brewer L. D. Dowling A. L. Curran-Rauhut M. A. Landfield P. W. Porter N. M. Blalock E. M. (2009). Estradiol reverses a calcium-related biomarker of brain aging in female rats. J. Neurosci. 29, 6058–6067. 10.1523/JNEUROSCI.5253-08.2009 19439583 PMC2740657

[B9] Brizzee K. R. Knox C. (1980). The aging process in the neuron. Adv. Exp. Med. Biol. 129, 71–98. 10.1007/978-1-4684-3734-8_9 6994458

[B10] Brody H. (1955). Organization of the cerebral cortex. III. A study of aging in the human cerebral cortex. J. Comp. Neurol. 102, 511–516. 10.1002/cne.901020206 14381544

[B11] Burke S. N. Barnes C. A. (2006). Neural plasticity in the ageing brain. Nat. Rev. Neurosci. 7, 30–40. 10.1038/nrn1809 16371948

[B12] Caetano M. S. Horst N. K. Harenberg L. Liu B. Arnsten A. F. Laubach M. (2012). Lost in transition: aging-related changes in executive control by the medial prefrontal cortex. J. Neurosci. 32, 3765–3777. 10.1523/JNEUROSCI.6011-11.2012 22423097 PMC3328309

[B13] Campbell L. W. Hao S. Y. Thibault O. Blalock E. M. Landfield P. W. (1996). Aging changes in voltage-gated calcium currents in hippocampal CA1 neurons. J. Neurosci. 16, 6286–6295. 8815908 10.1523/JNEUROSCI.16-19-06286.1996 PMC6579167

[B14] Chang Y.-M. Rosene D. L. Killiany R. J. Mangiamele L. A. Luebke J. I. (2005). Increased action potential firing rates of layer 2/3 pyramidal cells in the prefrontal cortex are significantly related to cognitive performance in aged monkeys. Cereb. Cortex 15, 409–418. 10.1093/cercor/bhh144 15749985

[B15] Chase M. H. Morales F. R. Boxer P. A. Fung S. J. (1985). Aging of motoneurons and synaptic processes in the cat. Exp. Neurol. 90, 471–478. 10.1016/0014-4886(85)90035-4 2996926

[B16] Chen K. S. Masliah E. Mallory M. Gage F. H. (1995). Synaptic loss in cognitively impaired aged rats is ameliorated by chronic human nerve growth factor infusion. Neuroscience 68, 19–27. 10.1016/0306-4522(95)00099-5 7477924

[B17] Chiu S. Y. (1991). Functions and distribution of voltage-gated sodium and potassium channels in mammalian Schwann cells. Glia 4, 541–558. 10.1002/glia.440040602 1720761

[B18] Coleman P. D. Flood D. G. (1987). Neuron numbers and dendritic extent in normal aging and Alzheimer’s disease. Neurobiol. Aging 8, 521–545. 10.1016/0197-4580(87)90127-8 3323927

[B19] de Brabander J. M. Kramers R. J. Uylings H. B. (1998). Layer-specific dendritic regression of pyramidal cells with ageing in the human prefrontal cortex. Eur. J. Neurosci. 10, 1261–1269. 10.1046/j.1460-9568.1998.00137.x 9749780

[B20] Disterhoft J. F. Oh M. M. (2006). Learning, aging and intrinsic neuronal plasticity. Trends Neurosci. 29, 587–599. 10.1016/j.tins.2006.08.005 16942805

[B21] Disterhoft J. F. Oh M. M. (2007). Alterations in intrinsic neuronal excitability during normal aging. Aging Cell 6, 327–336. 10.1111/j.1474-9726.2007.00297.x 17517042

[B22] Disterhoft J. F. Thompson L. T. Moyer J. R. Jr. Mogul D. J. (1996). Calcium-dependent afterhyperpolarization and learning in young and aging hippocampus. Life Sci. 59, 413–420. 10.1016/0024-3205(96)00320-7 8761329

[B23] Dodge F. A. Cooley J. W. (1973). Action potential of the Motorneuron. IBM J. Res. Dev. 17, 219–229 10.1147/rd.173.0219

[B24] Driver C. Georgiou A. Georgiou G. (2004). The contribution by mitochondrially induced oxidative damage to aging in *Drosophila melanogaster*. Biogerontology 5, 185–192. 10.1023/b:bgen.0000031156.75376.e3 15190188

[B25] Duan H. Wearne S. L. Rocher A. B. Macedo A. Morrison J. H. Hof P. R. (2003). Age-related dendritic and spine changes in corticocortically projecting neurons in macaque monkeys. Cereb. Cortex 13, 950–961. 10.1093/cercor/13.9.950 12902394

[B26] Erraji-Benchekroun L. Underwood M. D. Arango V. Galfalvy H. Pavlidis P. Smyrniotopoulos P.. (2005). Molecular aging in human prefrontal cortex is selective and continuous throughout adult life. Biol. Psychiatry 57, 549–558. 10.1016/j.biopsych.2004.10.034 15737671

[B27] Fieber L. A. Carlson S. L. Capo T. R. Schmale M. C. (2010). Changes in D-aspartate ion currents in the Aplysia nervous system with aging. Brain Res. 1343, 28–36. 10.1016/j.brainres.2010.05.001 20452331 PMC3062251

[B28] Foehring R. C. Surmeier D. J. (1993). Voltage-gated potassium currents in acutely dissociated rat cortical neurons. J. Neurophysiol. 70, 51–63. 8395588 10.1152/jn.1993.70.1.51

[B29] Foster T. C. (2007). Calcium homeostasis and modulation of synaptic plasticity in the aged brain. Aging Cell 6, 319–325. 10.1111/j.1474-9726.2007.00283.x 17517041

[B30] Gant J. C. Sama M. M. Landfield P. W. Thibault O. (2006). Early and simultaneous emergence of multiple hippocampal biomarkers of aging is mediated by Ca2+-induced Ca2+ release. J. Neurosci. 26, 3482–3490. 10.1523/jneurosci.4171-05.2006 16571755 PMC6673869

[B31] Gazzaley A. H. Thakker M. M. Hof P. R. Morrison J. H. (1997). Preserved number of entorhinal cortex layer II neurons in aged macaque monkeys. Neurobiol. Aging 18, 549–553. 10.1016/s0197-4580(97)00112-7 9390783

[B32] Giese K. P. Storm J. F. Reuter D. Fedorov N. B. Shao L. R. Leicher T.. (1998). Reduced K+ channel inactivation, spike broadening and after-hyperpolarization in Kvbeta1.1-deficient mice with impaired learning. Learn. Mem. 5, 257–273. 10454353 PMC311244

[B33] Gu N. Vervaeke K. Hu H. Storm J. F. (2005). Kv7/KCNQ/M and HCN/h, but not KCa2/SK channels, contribute to the somatic medium after-hyperpolarization and excitability control in CA1 hippocampal pyramidal cells. J. Physiol. 566, 689–715. 10.1113/jphysiol.2005.086835 15890705 PMC1464792

[B34] Harley P. R. (1975). A possible age-related decrement in the conduction velocity of Aplysia neuron R2. Experientia 31, 901–902. 10.1007/bf02358840 1157850

[B35] Hickmott P. Dinse H. (2013). Effects of aging on properties of the local circuit in rat primary somatosensory cortex (S1) in vitro. Cereb. Cortex 23, 2500–2513. 10.1093/cercor/bhs248 22879353

[B36] Hinman J. D. Peters A. Cabral H. Rosene D. L. Hollander W. Rasband M. N.. (2006). Age-related molecular reorganization at the node of Ranvier. J. Comp. Neurol. 495, 351–362. 10.1002/cne.20886 16485288 PMC4444368

[B37] Hsu K. S. Huang C. C. Liang Y. C. Wu H. M. Chen Y. L. Lo S. W.. (2002). Alterations in the balance of protein kinase and phosphatase activities and age-related impairments of synaptic transmission and long-term potentiation. Hippocampus 12, 787–802. 10.1002/hipo.10032 12542230

[B38] Hua T. Li X. He L. Zhou Y. Wang Y. Leventhal A. G. (2006). Functional degradation of visual cortical cells in old cats. Neurobiol. Aging 27, 155–162. 10.1016/j.neurobiolaging.2004.11.012 16298251

[B39] Iwamoto M. Hagishita T. Shoji-Kasai Y. Ando S. Tanaka Y. (2004). Age-related changes in the levels of voltage-dependent calcium channels and other synaptic proteins in rat brain cortices. Neurosci. Lett. 366, 277–281. 10.1016/j.neulet.2004.05.048 15288434

[B40] Jacobs B. Driscoll L. Schall M. (1997). Life-span dendritic and spine changes in areas 10 and 18 of human cortex: a quantitative Golgi study. J. Comp. Neurol. 386, 661–680. 10.1002/(sici)1096-9861(19971006)386:4<661::aid-cne11>3.0.co;2-n 9378859

[B41] Kadakkuzha B. M. Akhmedov K. Capo T. R. Carvalloza A. C. Fallahi M. Puthanveettil S. V. (2013). Age-associated bidirectional modulation of gene expression in single identified R15 neuron of Aplysia. BMC Genomics 14:880. 10.1186/1471-2164-14-880 24330282 PMC3909179

[B42] Kempsell A. T. Fieber L. A. (2014). Behavioral aging is associated with reduced sensory neuron excitability in Aplysia californica. Front. Aging Neurosci. 6:84. 10.3389/fnagi.2014.00084 24847260 PMC4023074

[B43] Kerr D. S. Campbell L. W. Hao S. Y. Landfield P. W. (1989). Corticosteroid modulation of hippocampal potentials: increased effect with aging. Science 245, 1505–1509. 10.1126/science.2781293 2781293

[B44] Keuker J. I. Luiten P. G. Fuchs E. (2003). Preservation of hippocampal neuron numbers in aged rhesus monkeys. Neurobiol. Aging 24, 157–165. 10.1016/s0197-4580(02)00062-3 12493561

[B45] Korngreen A. Sakmann B. (2000). Voltage-gated K+ channels in layer 5 neocortical pyramidal neurones from young rats: subtypes and gradients. J. Physiol. 525, 621–639. 10.1111/j.1469-7793.2000.00621.x 10856117 PMC2269970

[B46] Kumar A. Bodhinathan K. Foster T. C. (2009). Susceptibility to calcium dysregulation during brain aging. Front. Aging Neurosci. 1:2. 10.3389/neuro.24.002.2009 20552053 PMC2874411

[B47] Kumar A. Foster T. C. (2004). Enhanced long-term potentiation during aging is masked by processes involving intracellular calcium stores. J. Neurophysiol. 91, 2437–2444. 10.1152/jn.01148.2003 14762159

[B48] Kumar A. Foster T. C. (2007). “Neurophysiology of old neurons and synapses,” in Brain Aging: Models, Methods and Mechanisms, ed Riddle D. R. (Boca Raton, FL: CRC Press), Section III, Chapter 10.

[B49] Lancaster B. Adams P. R. (1986). Calcium-dependent current generating the afterhyperpolarization of hippocampal neurons. J. Neurophysiol. 55, 1268–1282. 2426421 10.1152/jn.1986.55.6.1268

[B50] Lancaster B. Nicoll R. A. (1987). Properties of two calcium-activated hyperpolarizations in rat hippocampal neurones. J. Physiol. 389, 187–203. 2445972 10.1113/jphysiol.1987.sp016653 PMC1192077

[B51] Landfield P. W. Pitler T. A. (1984). Prolonged Ca2+-dependent afterhyperpolarizations in hippocampal neurons of aged rats. Science 226, 1089–1092. 10.1126/science.6494926 6494926

[B52] Leventhal A. G. Wang Y. Pu M. Zhou Y. Ma Y. (2003). GABA and its agonists improved visual cortical function in senescent monkeys. Science 300, 812–815. 10.1126/science.1082874 12730605

[B53] Lu C. B. Hamilton J. B. Powell A. D. Toescu E. C. Vreugdenhil M. (2011). Effect of ageing on CA3 interneuron sAHP and gamma oscillations is activity-dependent. Neurobiol. Aging 32, 956–965. 10.1016/j.neurobiolaging.2009.05.006 19523715

[B54] Luebke J. I. Chang Y. M. (2007). Effects of aging on the electrophysiological properties of layer 5 pyramidal cells in the monkey prefrontal cortex. Neuroscience 150, 556–562. 10.1016/j.neuroscience.2007.09.042 17981400 PMC2410025

[B55] Luebke J. I. Chang Y. M. Moore T. L. Rosene D. L. (2004). Normal aging results in decreased synaptic excitation and increased synaptic inhibition of layer 2/3 pyramidal cells in the monkey prefrontal cortex. Neuroscience 125, 277–288. 10.1016/j.neuroscience.2004.01.035 15051166

[B56] Luebke J. I. Medalla M. Amatrudo J. M. Weaver C. M. Crimins J. L. Hunt B.. (2013). Age-Related changes to layer 3 pyramidal cells in the rhesus monkey visual cortex. Cereb. Cortex [Epub ahead of print]. 10.1093/cercor/bht336 24323499 PMC4428297

[B57] Luebke J. I. Rosene D. L. (2003). Aging alters dendritic morphology, input resistance and inhibitory signaling in dentate granule cells of the rhesus monkey. J. Comp. Neurol. 460, 573–584. 10.1002/cne.10668 12717715

[B58] Maccaferri G. Mangoni M. Lazzari A. DiFrancesco D. (1993). Properties of the hyperpolarization-activated current in rat hippocampal CA1 pyramidal cells. J. Neurophysiol. 69, 2129–2136. 7688802 10.1152/jn.1993.69.6.2129

[B59] Madison D. V. Nicoll R. A. (1984). Control of the repetitive discharge of rat CA 1 pyramidal neurones in vitro. J. Physiol. 354, 319–331. 6434729 10.1113/jphysiol.1984.sp015378 PMC1193414

[B60] Matthews E. A. Linardakis J. M. Disterhoft J. F. (2009). The fast and slow afterhyperpolarizations are differentially modulated in hippocampal neurons by aging and learning. J. Neurosci. 29, 4750–4755. 10.1523/JNEUROSCI.0384-09.2009 19369544 PMC2678237

[B61] Merrill D. A. Chiba A. A. Tuszynski M. H. (2001). Conservation of neuronal number and size in the entorhinal cortex of behaviorally characterized aged rats. J. Comp. Neurol. 438, 445–456. 10.1002/cne.1327 11559900

[B62] Merrill D. A. Roberts J. A. Tuszynski M. H. (2000). Conservation of neuron number and size in entorhinal cortex layers II, III and V/VI of aged primates. J. Comp. Neurol. 422, 396–401. 10.1002/1096-9861(20000703)422:3<396::aid-cne6>3.0.co;2-r 10861515

[B63] Morales F. R. Boxer P. A. Fung S. J. Chase M. H. (1987). Basic electrophysiological properties of spinal cord motoneurons during old age in the cat. J. Neurophysiol. 58, 180–194. 3612223 10.1152/jn.1987.58.1.180

[B64] Moroz L. L. Kohn A. B. (2010). Do different neurons age differently? Direct genome-wide analysis of aging in single identified cholinergic neurons. Front. Aging Neurosci. 2:6. 10.3389/neuro.24.006.2010 20725513 PMC2910937

[B65] Moyer J. R. Jr. Power J. M. Thompson L. T. Disterhoft J. F. (2000). Increased excitability of aged rabbit CA1 neurons after trace eyeblink conditioning. J. Neurosci. 20, 5476–5482. 10884331 10.1523/JNEUROSCI.20-14-05476.2000 PMC6772307

[B66] Moyer J. R. Jr. Thompson L. T. Black J. P. Disterhoft J. F. (1992). Nimodipine increases excitability of rabbit CA1 pyramidal neurons in an age- and concentration-dependent manner. J. Neurophysiol. 68, 2100–2109. 1491260 10.1152/jn.1992.68.6.2100

[B67] Need A. C. Irvine E. E. Giese K. P. (2003). Learning and memory impairments in Kv beta 1.1-null mutants are rescued by environmental enrichment or ageing. Eur. J. Neurosci. 18, 1640–1644. 10.1046/j.1460-9568.2003.02889.x 14511342

[B68] Oh M. M. Oliveira F. A. Disterhoft J. F. (2010). Learning and aging related changes in intrinsic neuronal excitability. Front. Aging Neurosci. 2:2. 10.3389/neuro.24.002.2010 20552042 PMC2874400

[B69] Page T. L. Einstein M. Duan H. He Y. Flores T. Rolshud D.. (2002). Morphological alterations in neurons forming corticocortical projections in the neocortex of aged Patas monkeys. Neurosci. Lett. 317, 37–41. 10.1016/s0304-3940(01)02428-4 11750991

[B70] Pakkenberg B. Gundersen H. J. (1997). Neocortical neuron number in humans: effect of sex and age. J. Comp. Neurol. 384, 312–320. 10.1002/(sici)1096-9861(19970728)384:2<312::aid-cne10>3.0.co;2-k 9215725

[B71] Peters A. (2002). The effects of normal aging on myelin and nerve fibers: a review. J. Neurocytol. 31, 581–593. 10.1023/A:1025731309829 14501200

[B72] Peters A. Leahu D. Moss M. B. McNally K. J. (1994). The effects of aging on area 46 of the frontal cortex of the rhesus monkey. Cereb. Cortex 4, 621–635. 10.1093/cercor/4.6.621 7703688

[B73] Peters A. Rosene D. L. Moss M. B. Kemper T. L. Abraham C. R. Tigges J.. (1996). Neurobiological bases of age-related cognitive decline in the rhesus monkey. J. Neuropathol. Exp. Neurol. 55, 861–874. 10.1097/00005072-199608000-00001 8759775

[B74] Peters A. Sethares C. Killiany R. J. (2001). Effects of age on the thickness of myelin sheaths in monkey primary visual cortex. J. Comp. Neurol. 435, 241–248. 10.1002/cne.1205 11391644

[B75] Peters A. Sethares C. Moss M. B. (1998). The effects of aging on layer 1 in area 46 of prefrontal cortex in the rhesus monkey. Cereb. Cortex 8, 671–684. 10.1093/cercor/8.8.671 9863695

[B76] Post-Munson D. J. Lum-Ragan J. T. Mahle C. D. Gribkoff V. K. (1994). Reduced bicuculline response and GABAA agonist binding in aged rat hippocampus. Neurobiol. Aging 15, 629–633. 10.1016/0197-4580(94)00057-3 7824055

[B77] Power J. M. Wu W. W. Sametsky E. Oh M. M. Disterhoft J. F. (2002). Age-related enhancement of the slow outward calcium-activated potassium current in hippocampal CA1 pyramidal neurons in vitro. J. Neurosci. 22, 7234–7243. 12177218 10.1523/JNEUROSCI.22-16-07234.2002 PMC6757904

[B78] Randall A. D. Booth C. Brown J. T. (2012). Age-related changes to Na+ channel gating contribute to modified intrinsic neuronal excitability. Neurobiol. Aging 33, 2715–2720. 10.1016/j.neurobiolaging.2011.12.030 22284989

[B79] Rapp P. R. Gallagher M. (1996). Preserved neuron number in the hippocampus of aged rats with spatial learning deficits. Proc. Natl. Acad. Sci. U S A 93, 9926–9930. 10.1073/pnas.93.18.9926 8790433 PMC38531

[B80] Rasband M. N. (2004). It’s “juxta” potassium channel! J. Neurosci. Res. 76, 749–757. 10.1002/jnr.20073 15160387

[B81] Rasmussen T. Schliemann T. Sorensen J. C. Zimmer J. West M. J. (1996). Memory impaired aged rats: no loss of principal hippocampal and subicular neurons. Neurobiol. Aging 17, 143–147. 10.1016/0197-4580(95)02032-2 8786797

[B82] Rogers J. Zornetzer S. F. Bloom F. E. (1981). Senescent pathology of cerebellum: Purkinje neurons and their parallel fiber afferents. Neurobiol. Aging 2, 15–25. 10.1016/0197-4580(81)90054-3 6267492

[B83] Rosene D. Nicholson T. (1999). “Neurotransmitter receptor changes in the hippocampus and cerebral cortex in normal aging,” in Cerebral Cortex, eds Peters A. Morrison J. (New York, Philadelphia: Springer), 111–128.

[B84] Sah P. (1996). Ca(2+)-activated K+ currents in neurones: types, physiological roles and modulation. Trends Neurosci. 19, 150–154. 10.1016/s0166-2236(96)80026-9 8658599

[B85] Sah P. Faber E. S. (2002). Channels underlying neuronal calcium-activated potassium currents. Prog. Neurobiol. 66, 345–353. 10.1016/s0301-0082(02)00004-7 12015199

[B86] Schmolesky M. T. Wang Y. Pu M. Leventhal A. G. (2000). Degradation of stimulus selectivity of visual cortical cells in senescent rhesus monkeys. Nat. Neurosci. 3, 384–390. 10.1038/73957 10725929

[B87] Sesti F. Liu S. Cai S. Q. (2010). Oxidation of potassium channels by ROS: a general mechanism of aging and neurodegeneration? Trends Cell Biol. 20, 45–51. 10.1016/j.tcb.2009.09.008 19850480

[B88] Shao L. R. Halvorsrud R. Borg-Graham L. Storm J. F. (1999). The role of BK-type Ca2+-dependent K+ channels in spike broadening during repetitive firing in rat hippocampal pyramidal cells. J. Physiol. 521, 135–146. 10.1111/j.1469-7793.1999.00135.x 10562340 PMC2269638

[B89] Sloane J. A. Hinman J. D. Lubonia M. Hollander W. Abraham C. R. (2003). Age-dependent myelin degeneration and proteolysis of oligodendrocyte proteins is associated with the activation of calpain-1 in the rhesus monkey. J. Neurochem. 84, 157–168. 10.1046/j.1471-4159.2003.01541.x 12485412

[B90] Stocker M. Krause M. Pedarzani P. (1999). An apamin-sensitive Ca2+-activated K+ current in hippocampal pyramidal neurons. Proc. Natl. Acad. Sci. U S A 96, 4662–4667. 10.1073/pnas.96.8.4662 10200319 PMC16389

[B91] Storm J. F. (1990). Potassium currents in hippocampal pyramidal cells. Prog. Brain Res. 83, 161–187. 10.1016/s0079-6123(08)61248-0 2203097

[B92] Sugiyama I. Tanaka K. Akita M. Yoshida K. Kawase T. Asou H. (2002). Ultrastructural analysis of the paranodal junction of myelinated fibers in 31-month-old-rats. J. Neurosci. Res. 70, 309–317. 10.1002/jnr.10386 12391590

[B93] Tanaka Y. Ando S. (2001). Age-related changes in the subtypes of voltage-dependent calcium channels in rat brain cortical synapses. Neurosci. Res. 39, 213–220. 10.1016/s0168-0102(00)00212-1 11223467

[B94] Thibault O. Hadley R. Landfield P. W. (2001). Elevated postsynaptic [Ca2+]i and L-type calcium channel activity in aged hippocampal neurons: relationship to impaired synaptic plasticity. J. Neurosci. 21, 9744–9756. 11739583 10.1523/JNEUROSCI.21-24-09744.2001 PMC6763040

[B95] Thibault O. Landfield P. W. (1996). Increase in single L-type calcium channels in hippocampal neurons during aging. Science 272, 1017–1020. 10.1126/science.272.5264.1017 8638124

[B96] Villalobos C. Shakkottai V. G. Chandy K. G. Michelhaugh S. K. Andrade R. (2004). SKCa channels mediate the medium but not the slow calcium-activated afterhyperpolarization in cortical neurons. J. Neurosci. 24, 3537–3542. 10.1523/jneurosci.0380-04.2004 15071101 PMC6729743

[B97] Wang M. Gamo N. J. Yang Y. Jin L. E. Wang X. J. Laubach M.. (2011). Neuronal basis of age-related working memory decline. Nature 476, 210–213. 10.1038/nature10243 21796118 PMC3193794

[B98] West M. J. (1993). New stereological methods for counting neurons. Neurobiol. Aging 14, 275–285. 10.1016/0197-4580(93)90112-o 8367009

[B99] West M. J. Coleman P. D. Flood D. G. Troncoso J. C. (1994). Differences in the pattern of hippocampal neuronal loss in normal ageing and Alzheimer’s disease. Lancet 344, 769–772. 10.1016/s0140-6736(94)92338-8 7916070

[B100] Wilson I. A. Ikonen S. Gallagher M. Eichenbaum H. Tanila H. (2005). Age-associated alterations of hippocampal place cells are subregion specific. J. Neurosci. 25, 6877–6886. 10.1523/jneurosci.1744-05.2005 16033897 PMC6725350

[B101] Wong T. P. Campbell P. M. Ribeiro-da-Silva A. Cuello A. C. (1998). Synaptic numbers across cortical laminae and cognitive performance of the rat during ageing. Neuroscience 84, 403–412. 10.1016/s0306-4522(97)00485-5 9539212

[B102] Wong T. P. Marchese G. Casu M. A. Ribeiro-da-Silva A. Cuello A. C. De Koninck Y. (2000). Loss of presynaptic and postsynaptic structures is accompanied by compensatory increase in action potential-dependent synaptic input to layer V neocortical pyramidal neurons in aged rats. J. Neurosci. 20, 8596–8606. 11069968 10.1523/JNEUROSCI.20-22-08596.2000 PMC6773180

[B103] Xi M. C. Liu R. H. Engelhardt J. K. Morales F. R. Chase M. H. (1999). Changes in the axonal conduction velocity of pyramidal tract neurons in the aged cat. Neuroscience 92, 219–225. 10.1016/s0306-4522(98)00754-4 10392844

[B104] Xing Y. Samuvel D. J. Stevens S. M. Dubno J. R. Schulte B. A. Lang H. (2012). Age-related changes of myelin basic protein in mouse and human auditory nerve. PLoS One 7:e34500. 10.1371/journal.pone.0034500 22496821 PMC3320625

[B105] Yan J. Zhang Y. Roder J. Mcdonald R. J. (2003). Aging effects on spatial tuning of hippocampal place cells in mice. Exp. Brain Res. 150, 184–193. 10.1007/s00221-003-1396-6 12677315

